# Integrating Color and Contour Analysis with Deep Learning for Robust Fire and Smoke Detection

**DOI:** 10.3390/s25072044

**Published:** 2025-03-25

**Authors:** Abror Shavkatovich Buriboev, Akmal Abduvaitov, Heung Seok Jeon

**Affiliations:** 1 Department of AI-Software, Gachon University, Seongnam-si 13120, Republic of Korea; abror1989@gachon.ac.kr; 2 Department of IT, Samarkand Branch of Tashkent University of Information Technologies, Samarkand 100084, Uzbekistan; akmalabduvaitov@gmail.com; 3 Department of Computer Engineering, Konkuk University, Chungju 27478, Republic of Korea

**Keywords:** concatenated CNN, fire and smoke detection, color and contour analysis

## Abstract

Detecting fire and smoke is essential for maintaining safety in urban, industrial, and outdoor settings. This study suggests a unique concatenated convolutional neural network (CNN) model that combines deep learning with hybrid preprocessing methods, such as contour-based algorithms and color characteristics analysis, to provide reliable and accurate fire and smoke detection. A benchmark dataset with a variety of situations, including dynamic surroundings and changing illumination, the D-Fire dataset was used to assess the technique. Experiments show that the suggested model outperforms both conventional techniques and the most advanced YOLO-based methods, achieving accuracy (0.989) and recall (0.983). In order to reduce false positives and false negatives, the hybrid architecture uses preprocessing to enhance Regions of Interest (ROIs). Additionally, pooling and fully linked layers provide computational efficiency and generalization. In contrast to current approaches, which frequently concentrate only on fire detection, the model’s dual smoke and fire detection capabilities increase its adaptability. Although preprocessing adds a little computing expense, the methodology’s excellent accuracy and resilience make it a dependable option for safety-critical real-world applications. This study sets a new standard for smoke and fire detection and provides a route forward for future developments in this crucial area.

## 1. Introduction

In urban, industrial, and natural settings, where early warning systems are critical to averting disastrous outcomes, fire and smoke detection is vital to maintaining safety. Reliable fire and smoke detection systems are urgently needed, since fires in places like woods, industrial complexes, and residential areas can result in a large loss of life and property. Because of their simplicity, traditional fire and smoke detection methods—such as color-based approaches and contour analysis—have been widely employed. However, they frequently fall short in addressing the difficulties presented by dynamic surroundings, changing lighting conditions, and overlapping fire and smoke characteristics. Similar to this, cutting-edge deep learning-based techniques like YOLO [1,2,3] and other convolutional neural networks [4,5,6,7,8,9] have shown impressive gains in speed and accuracy, but they are still constrained by their reliance on feature extraction that is solely driven by data, which can result in missed detections and false positives in complex situations.

By cutting down on false alarms and reaction times, the use of sophisticated fire and smoke detection systems is essential for improving safety and resource management. Because they work well in a variety of settings, traditional sensor-based techniques, including heat, smoke, flame, and gas sensors, have been used extensively in fire detection systems. These systems have demonstrated dependability in several applications, as detailed in papers [10,11,12,13,14,15,16]. They do, however, have innate constraints that affect how well they operate overall. For example, smoke sensors are prone to false alarms caused by particles unrelated to fire, while heat sensors frequently show delayed reactions in quickly expanding flames.

Researchers are increasingly using machine learning techniques to analyze visual data, including color, texture, and motion, for fire detection in order to overcome these constraints. For instance, color-based models use the unique color characteristics of flames to distinguish fire pixels. Although these models have demonstrated potential, as noted in [17,18,19,20,21,22,23,24,25,26], they frequently perform poorly in different lighting circumstances or when items with colors similar to flames are present.

Even though machine learning techniques have advanced, their use still necessitates thorough preprocessing and feature extraction procedures to address issues including changing situations, overlapping fire and smoke variables, and computing limitations. These techniques are frequently computationally demanding and may not work well in real-time applications, especially when safety is at stake. In order to overcome these obstacles, a more dependable and effective fire and smoke detection system that integrates sophisticated deep learning architectures with strong preprocessing approaches is obviously required.

This work combines the advantages of sophisticated deep learning architectures and conventional preprocessing techniques to offer a unique concatenated CNN model that addresses the shortcomings of current approaches. The hybrid method combines CNNs with contour-based algorithms and color characteristics analysis to improve the accuracy of feature extraction and classification. The model more successfully separates smoke and fire areas by utilizing preprocessing techniques, which lowers the number of false alarms brought on by visually similar things like sunlight reflections or bright clothes. Furthermore, the deep learning architecture of the model was improved using dropout mechanisms to avoid overfitting, fully linked layers to capture pertinent information, and pooling layers to reduce dimensionality. The D-Fire dataset [27], a comprehensive dataset with a variety of situations, including changing lighting conditions (day and night), dynamic surroundings (explosions and burning automobiles), and complicated settings (interior and outdoor sceneries), was used to assess the suggested model. The findings show that the suggested concatenated CNN model outperforms state-of-the-art techniques like YOLOv8, Pruned YOLOv4, and GS-YOLOv5 by achieving precision (0.989) and recall (0.983). In addition to reducing false positives and false negatives, this hybrid approach improves the system’s capacity to identify smoke and fire, increasing its adaptability and dependability for practical uses.

The main contributions of this study are as follows:

Hybrid architecture for fire and smoke detection: The development of a concatenated CNN model that integrates traditional preprocessing techniques (color characteristics analysis and contour-based algorithms) with deep learning, addressing the limitations of purely data-driven or handcrafted methods.

Dual detection capability: The proposed model effectively detects both fire and smoke, making it more versatile than existing methods that focus solely on fire detection.

Enhanced feature extraction: By leveraging preprocessing techniques to refine Regions of Interest (ROIs), the model reduces noise and isolates fire and smoke regions, improving the accuracy of classification and reducing false positives.

Superior performance on the D-Fire dataset: The model achieves precision (0.989) and recall (0.983) on the D-Fire dataset, outperforming state-of-the-art YOLO-based approaches, and demonstrates robustness across diverse scenarios, including varying lighting, dynamic environments, and complex overlapping features.

The methodology’s minor computing burden from preprocessing, despite its great accuracy and durability, makes it less appropriate for severely limited real-time applications without further optimization. Future research might concentrate on expanding the system to manage real-time streaming data and lowering computing complexity without sacrificing accuracy.

The structure of this document is as follows: A thorough summary of relevant research and current fire and smoke detection limits is given in Section 2. The suggested technique, including the CNN framework and hybrid preprocessing, is explained in Section 3. The experimental findings and their interpretation are shown in Section 4, which also contrasts the suggested model with cutting-edge techniques. The study is concluded in Section 5 with a summary of the results and a discussion of possible directions for further investigation.

## 2. Related Works

The development of smoke and fire detection devices has benefited greatly from both conventional and deep learning-based methods. With an emphasis on techniques, datasets, and the development of detection systems, this section examines current and significant research in the subject. The framework for the creation of the suggested hybrid concatenated CNN model is established by the study, which also highlights the innovations and shortcomings of current approaches. Sultan et al. [28] presented a deep learning-based multistage fire detection system that successfully detected fires by combining deep networks and preprocessing methods. Although their method showed higher detection rates, its real-time applications were limited by its computing complexity due to its reliance on multistage processing. In a similar vein, Gonçalves et al. [29] investigated YOLO-based models for identifying smoke and wildfires in aerial and ground data, with consistent outcomes across a range of settings. Higher false positives resulted from the end-to-end YOLO architecture’s difficulties in situations when fire and smoke characteristics overlapped.

A multi-attention network was used by Khan et al. [30] to improve real-time fire detection by improving detection precision and fine-tuning feature extraction. Their study significantly advanced the discipline by introducing a benchmark dataset for fire detection. Even with these improvements, their network was not designed to detect smoke, which restricts its use in situations when smoke serves as the main signal. In their study on marine safety, Ergasheva et al. [31] employed histogram equalization, deep learning, and computer vision to identify ship fires. Despite its success in the marine field, the approach was less applicable in other settings due to its dependence on histogram-based preprocessing. A fire and smoke detection system based on dilated CNNs was presented by Valikhujaev et al. [32], who showed its outstanding performance in surveillance applications. However, scalability for real-time applications was limited due to their dependence on dilated convolutions, which raised computational expenses. Kim and Muminov [33] used deep learning and photos from unmanned aerial vehicles (UAVs) to identify smoke from forest fires. Although this method worked quite well in large-scale outdoor settings, it had trouble generalizing to interior or mixed contexts. Choueiri et al. [34] used artificial neural networks (ANNs) to detect smoke and fire, with encouraging outcomes in controlled settings. Their model, however, had trouble in dynamic environments when background objects had visual characteristics that were akin to smoke or fire. Using cutting-edge deep learning approaches, Chitram et al. [35] suggested improvements in smoke and fire detection. They highlighted increases in model accuracy but did not address the problem of false positives brought on by things that resemble fire.

Namozov and Cho’s [36] effective deep learning method produced dependable smoke and fire detection with fewer data. Despite helping low-resource applications, this method was not scalable to bigger datasets or real-world settings. Similar to this, Sathishkumar et al. [37] used a “learning without forgetting” paradigm to solve the difficulties of continuous learning in smoke and fire detection; however, they had trouble incorporating a variety of scenarios into their training. For automatic fire detection, Dogan et al. [38] suggested an ensemble pretrained residual network, which achieved good accuracy in controlled settings. However, the computational burden that ensemble techniques frequently incur restricts their use in real-time systems. Hu et al. [39] used MVMNet to create a rapid detection framework for smoke detection from forest fires. Despite achieving real-time performance, their model was not strong enough to recognize smoke and fire characteristics that overlapped. For fire detection, Jeon et al. [40] developed a multi-scale prediction CNN that increased accuracy in situations with different object sizes but fell short in smoke detection. A motion-based fire/smoke detection technique in conjunction with CNNs was proposed by Luo et al. [41]. It showed high performance for video-based detection systems but limited application for static picture detection. Their work showcased the adaptability of these models to various environments and the ability to achieve precise detection outcomes.

Although the aforementioned research shows notable progress in smoke and fire detection, a number of restrictions still exist:

- Smoke detection: Many techniques, especially those based on YOLO, are mainly concerned with detecting fires and have little ability to detect smoke.
- False positives: Fire-like items or overlapping fire and smoke characteristics frequently lead models to have high false-alert rates.
- Scalability: Approaches that depend on computationally costly methods, such as dilated convolutions or ensemble networks, have trouble expanding to real-time applications.
- Dataset limitations: Few models, especially those trained on small datasets or specialized contexts, generalize effectively across a variety of scenarios.

The suggested concatenated CNN model expands on these discoveries and overcomes their drawbacks by combining deep learning and conventional preprocessing methods in a hybrid architecture. In order to improve smoke detection capabilities and minimize false positives, the suggested model makes use of color features and contour analysis for preprocessing. Additionally, as compared to the studied approaches, its application to the D-Fire dataset, which includes a variety of scenarios, guarantees higher generalization and scalability. In safety-critical settings, this hybrid method validates the suggested model as a solid and dependable solution for smoke and fire detection.

## 3. Materials and Methods

**Figure 1:**
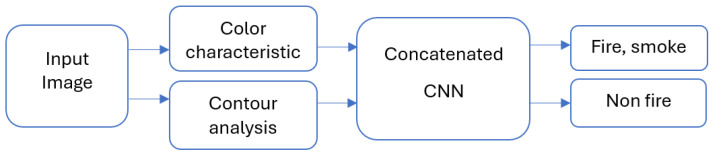
Overall block-scheme of proposed model.

The proposed concatenated CNN model combines color characteristics analysis and contour analysis with a deep learning framework to achieve robust fire and smoke detection. As shown in Figure 1, the method consists of the following stages:

- Preprocessing to extract color and contour features;
- Feature concatenation and processing through a CNN;
- Classification of the input image as fire, smoke, or non-fire.

Each stage is described in detail below.

**Input image**: The procedure starts with an input image, which can be taken from pre-existing datasets or in real-time by security cameras. These pictures could have smoke, fire, or other visual components in a variety of lighting and environmental settings. The hybrid model is intended to effectively evaluate these inputs and derive significant features for the classification of smoke and fire. Two parallel branches scan the input image to extract important features to improve detection accuracy:

**Color characteristics**: This block examines the input image’s possible fire characteristics, paying particular attention to areas that exhibit fire-like traits. It chooses Regions of Interest (ROIs) according to fire-typical color characteristics.

Fire is known for its red, orange, and yellow hues, which are frequently very vivid and saturated. Smoke is usually white or gray, but the color can change based on what is burning (e.g., light smoke for vegetation fires or dark smoke for industrial fires).

These characteristics offer a solid basis for differentiating smoke and fire from other substances, like clouds or reflections of sunlight.

**Contour analysis**: This block uses contours to investigate the geometric and structural characteristics of possible fire or smoke zones while working simultaneously with the color characteristics. The following criteria are used to examine contours:

- Area—removes areas that are either too big or too small to depict fire.
- Aspect ratio—detects asymmetrical forms that are characteristic of smoke or flames.
- Convexity—identifies the flickering or curving lines that delineate fire zones.

The contour analysis block eliminates false positives from non-fire features while ensuring that the ROIs match realistic fire or smoke patterns by combining these filters.

**Concatenated CNN**: The central component of the system, a concatenated CNN, receives the characteristics that were derived from the color characteristics and contour analysis blocks. Concatenated CNNs are a kind of neural network architecture that improves prediction accuracy by combining features from several sources or modalities. Here, the contour-based and color-based characteristics are combined to create a single, cohesive representation. These inputs teach the CNN hierarchical feature representations, which enable it to recognize intricate patterns and connections that differentiate smoke and fire from non-fire components. The system can manage a variety of situations thanks to this structure, such as overlapping fire and smoke, changing lighting, and unclear patterns brought on by outside influences.

**Output**: The model generates a classification result after running the features through the concatenated CNN, classifying the input image into one of two groups.

### 3.1. Dataset

**Figure 2:**
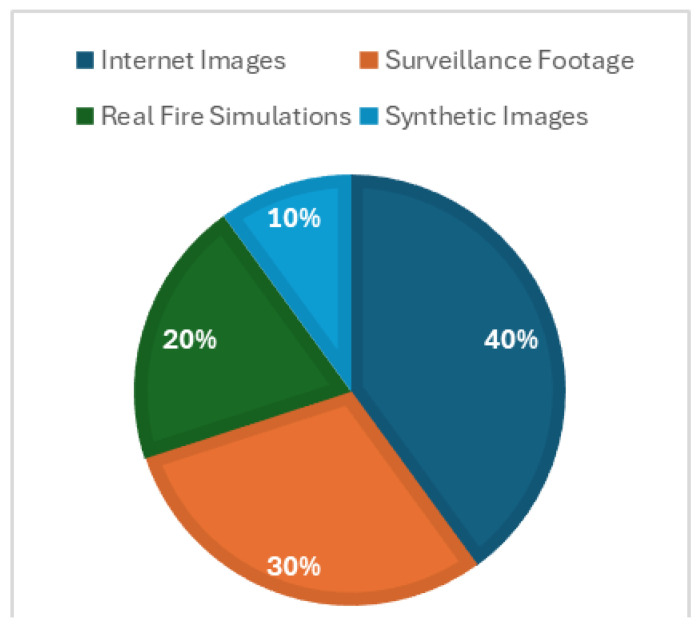
Composition of the D-Fire dataset.

The D-Fire dataset, publicly available on Kaggle (https://www.kaggle.com/datasets/shubhamkarande13/d-fire accessed on 23 March 2025) [27], comprises 21,527 images designed for smoke and fire detection applications. These are categorized into three classes: “fire” (only fire), “smoke” (only smoke), and “fire and smoke” (both fire and smoke). The dataset includes 26,557 bounding boxes, with 14,692 labeled as fire and 11,865 as smoke. D-Fire is diverse, sourced from multiple origins: internet images, real fire simulations, and surveillance footage from Serra Verde State Park and the Universidad Federal de Minas Gerais (UFMG), Brazil. The composition and source pathway of the D-Fire dataset from Kaggle is illustrated in Figure 2, showing the estimated distribution across internet images (40%, 8611 images), surveillance footage (30%, 6458 images), real fire simulations (20%, 4305 images), and synthetic images (10%, 2153 images), as provided by the dataset creators.

Additionally, the dataset incorporates synthetic images with artificial smoke overlaid on real-world scenes, a feature provided by the D-Fire dataset creators to enhance robustness, as described on the Kaggle page. No additional augmentation was performed in this study.

For training, validating, and testing the proposed concatenated CNN model, the dataset of 21,527 images was split into a 70/15/15 ratio, resulting in 15,069 images for the training set, 3229 images for the validation set, and 3229 images for the test set. This split ensured a balanced approach to model development, allowing for robust training, effective hyperparameter tuning, and unbiased performance evaluation.

This dataset presents significant challenges due to variations in fire and smoke patterns, lighting conditions, and camera angles. Factors such as wind direction, combustion materials, and fire size further contribute to detection complexity. The comprehensive annotations and scenario diversity, encompassing dynamic environments (e.g., explosions and burning cars), varied lighting conditions (day/night), and complex settings (indoor/outdoor), make D-Fire a valuable benchmark for fire and smoke detection research.

### 3.2. Color Characteristics and Contour Analysis Methods

**Figure 3:**
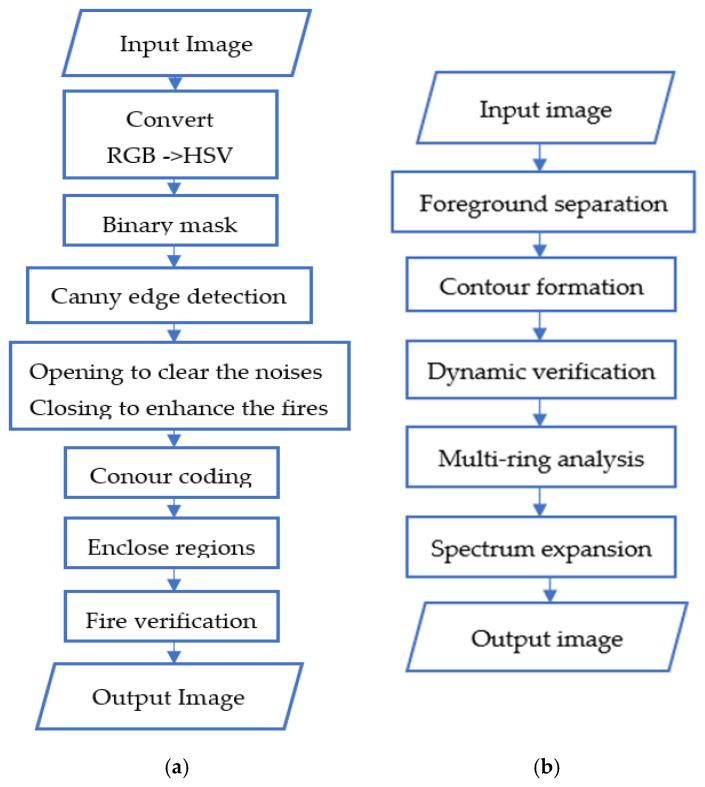
Overall block diagram: (**a**) color characteristics; (**b**) contour analysis methods.

The suggested fire and smoke detection model combines color-based and contour-based techniques with the following goals: minimizing false positives, increasing detection accuracy, speeding up neural network processing, and reducing image size by choosing Regions of Interest (ROIs), as shown in Figure 3. Utilizing the complimentary advantages of these methods, the system performs reliably and effectively in a range of environmental settings, making it ideal for real-world fire monitoring and detection applications.

Color-based fire and smoke detection is one of the oldest and most popular methods. The procedure entails examining the unique color characteristics which set fire and smoke apart from most other objects in ordinary settings. The following are the procedures for identifying fire based on color characteristics:

Step 1: Convert to HSV color space—the RGB image is converted to HSV for better segmentation of fire hues (red, orange, and yellow). This separation enhances robustness to lighting variations and simplifies contour detection.

Step 2: Create a binary mask—a mask is applied to isolate fire-like regions based on predefined HSV thresholds (upperBound = [145, 255, 255], lowerBound = [0, 0, 200]).

Step 3: Edge detection—use the canny edge detector to identify fire boundaries.

Step 4: Morphological operations—apply opening to remove noise and closing to enhance fire regions.

Step 5: Contour coding—the boundaries of the detected fire regions are encoded using the Freeman chain code method, which is invariant to displacement, rotation, and scale.

Step 6: Frame contours—the detected contours are enclosed in rectangular frames and labeled for further analysis.

Step 7: Flame verification—the algorithm checks if the detected regions meet the criteria for being classified as flames (e.g., size, intensity, and distribution).

The procedures for identifying smoke based on color characteristics:

Step 1: The RGB image is converted to HSV to segment smoke-like regions, targeting gray-to-white hues with moderate saturation.

Step 2: Create a binary mask—a mask is applied using HSV thresholds (lowerBound = [0, 0, 150], upperBound = [180, 50, 255]) to isolate smoke regions, accommodating variations due to combustion materials.

Step 3: Edge detection—the canny edge detector identifies smoke boundaries, adjusted for diffuse edges.

Step 4: Morphological operations—opening removes noise, while closing enhances the continuity of smoke plumes.

Step 5: Contour coding—smoke boundaries are encoded using the Freeman chain code, adapted for irregular shapes.

Step 6: Frame contours—contours are framed and labeled for analysis.

Step 7: Smoke verification—the algorithm verifies smoke presence based on area, intensity variations, and diffusion patterns.

Fire and smoke detection relying solely on color may provide false positives because of similar-colored objects (e.g., sunsets, fogs, and yellow foliage). The purpose of contour-based analysis is to increase the accuracy of detection. The following are the steps for detecting fires using contours:

Step 1: Foreground separation—perform raster binarization to isolate fire objects from the background. Let R = {r_m,n_} m = $\bar{1,M}$, where n = $\bar{1,N}—$represent an image containing a separate dynamic object; then, the rule for binarizing this image will look like the following:

$${\hat{r}}_{m,n}=\left\{\begin{array}{c} 0\;if\;r_{m,n}\leq \beta \\ 255\;if\;r_{m,n}>\beta \; \end{array}\right\}$$

where $\hat{R}=\{{\hat{r}}_{m,n}\}$ is a binarized image; β is a threshold value, which is selected based on the histogram of pixel brightness distribution H-{h_k_}; and k = $\bar{0,\;255}$ is the current raster. Pixels are classified into two classes: object and background.

Step 2: Contour formation—the Beetle algorithm is used to track the boundaries of the fire region. A “beetle” moves along the boundary of the object, turning left or right to follow the contour. The output is a contour represented as a vector of complex numbers.

Step 3: Dynamic verification—the algorithm checks the intensity of the inner region of the contour to ensure that it corresponds to fire. Stochastic movements of the fire region are analyzed to confirm its dynamic nature.

Step 4: Multi-ring analysis—identify regions as inner and outer ring concentric zones.

Step 5: Spectrum expansion—use a Gaussian distribution to refine contours in the HSV palette.

Smoke often appears as a lighter, less intense region (gray to white) with varying opacity, blending into the background more gradually. This suggests that the threshold *β* should be adjusted to capture lower-intensity pixels and account for smoke’s diffuse nature. The histogram analysis should focus on identifying a brightness range where smoke pixels dominate, typically in the mid-to-high brightness levels but with lower saturation compared to fire. The smoke detection using the contour analysis is as follows:

Step 1: Foreground separation—raster binarization isolates smoke objects, with a threshold β = 150, adjusted for lower contrast.

Step 2: Contour formation—the Beetle algorithm tracks smoke boundaries, focusing on diffuse, irregular shapes.

Step 3: Dynamic verification—intensity variations and spreading patterns confirm smoke presence.

Step 4: Multi-ring analysis—identifies concentric zones for larger, amorphous smoke regions.

Step 5: Spectrum expansion—a Gaussian distribution refines smoke contours, enhancing detection in varied lighting.

The integration of color characteristics and contour-based detection provides a robust framework for fire and smoke detection. Color analysis identifies potential fire and smoke regions based on hue and intensity. Contour analysis refines detection by verifying fire-like boundaries and dynamic movement. Together, these methods reduce false positives and enhance accuracy, ensuring reliable performance in diverse environments. The combined algorithms ensure that fire is accurately detected even in challenging conditions, such as fluctuating lighting, overlapping objects, and dynamic movements, making them ideal for real-world applications like surveillance systems and early warning systems.

### 3.3. Architecture of the Concatenated CNN Model

**Figure 4:**
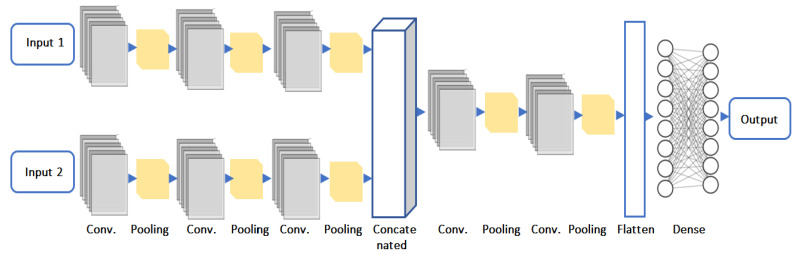
Architecture of proposed CCNN model.

The architecture of the suggested concatenated CNN model, intended for precise fire and smoke detection, is depicted in Figure 4. To distinguish between fire, smoke, and non-fire elements, this model combines data from two inputs, processes them through deep learning layers, and then outputs classifications. The model’s components are explained in detail below:

**Input 1**: Features taken from the color characteristics analyzer, which uses color attributes, including hue, saturation, brightness, and intensity gradients, to find areas of the image that resemble fire.

**Input 2**: Features from the contour analysis, which identifies possible fire or smoke zones using structural and geometric properties, including boundaries, area, aspect ratio, and convexity.

Each input is processed independently through the model’s respective branches to ensure that both feature sets are adequately analyzed.

**Convolution layers**:

- First convolutional layer: Applies 64 filters with a 3 × 3 kernel size, and then an activation function called ReLU. The primary objective of this layer is to extract fundamental elements from the input images, like edges and simple textures. The size of the feature map in output is 126 × 126 × 64.
- Second convolutional layer: Applies 128 filters, each having a 3 × 3 kernel, and then the ReLU activation function. By expanding on the results of the previous layer, this layer can capture more intricate details. Because of max pooling, the feature map’s size is lowered to 62 × 62 × 128.
- Third convolutional layer: Applies an ReLU activation function after 256 filters with a 3 × 3 kernel size. This layer further abstracts the visual data by identifying high-level properties like certain patterns associated with fire. After pooling, the feature map measures 30 × 30 × 256.

Prior to merging, these distinct branches guarantee that color-based and contour-based characteristics are learned independently and as efficiently as possible.

**Concatenated features**: At this stage, the outputs from both input branches are concatenated along the final axis, forming a combined 64 × 64 × 768 feature map (256 from each branch). This step integrates diverse feature representations, enhancing the model’s ability to distinguish fire and smoke patterns effectively.

**Post-concatenated hidden layers**: The concatenated feature map is then processed by the fourth convolutional layer, which applies 512 filters with a 3 × 3 kernel and ReLU activation, refining the extracted patterns. A 2 × 2 pooling operation reduces the feature map size to 32 × 32 × 512. The fifth and final convolutional layer employs 1024 filters with a 3 × 3 kernel, capturing highly abstract features. The subsequent 2 × 2 pooling layer further downsamples the feature map to 16 × 16 × 1024, preparing it for classification. To transition from convolutional layers to fully connected layers, a flatten layer converts the 3D feature map into a 1D vector. The first dense layer consists of 1024 neurons with ReLU activation, followed by a dropout layer with a rate of 0.5 to prevent overfitting by randomly deactivating 50% of neurons during training. A second dense layer with 512 neurons further refines the feature representations, followed by another dropout layer (rate = 0.5) to improve generalization.

**Output layer**: The output layer employs a Softmax activation function with two neurons, producing probability scores for the classes: fire, smoke, or non-fire. See Table 1.

The suggested concatenated CNN model is a hybrid model that successfully blends the advantages of deep learning and image processing, guaranteeing precise and trustworthy smoke and fire detection in challenging, real-world situations.

### 3.4. Training Setup Parameters

The suggested concatenated CNN model’s training parameters were carefully selected to maximize performance in smoke and fire detection tasks. By maintaining a balance between detection accuracy and computing economy, these parameters enable the model to learn efficiently without overfitting.

The loss function used by the model is Binary Cross-Entropy, which works well for binary classification tasks. By minimizing the discrepancy between the actual class labels and the projected probabilities, this loss function directs the model to iteratively improve its predictions. With a starting learning rate of 0.001, the Adam optimizer is employed to maximize the learning process. Adam is well known for its effectiveness and versatility, fusing the benefits of adjustable learning rates and momentum to converge more quickly and manage challenging datasets.

Here are the training parameters of the proposed CNN model:

- Loss Function: Binary Cross-Entropy, ideal for binary classification tasks.
- Optimizer: Adam optimizer with an initial learning rate of 0.001, known for its efficiency and adaptability.
- Batch Size: Thirty-two, balancing computational load and convergence speed.
- Number of Epochs: Fifty, ensuring sufficient learning while minimizing overfitting risks.
- Early Stopping: Implemented to monitor the validation loss, with a patience parameter set to 10 epochs, stopping training when the validation loss does not improve.

Complex features are extracted and combined from the input data using the concatenated CNN’s architecture. The model may take use of complementing visual and structural information by combining features from two different inputs: color characteristics and contour analysis. By reducing dimensionality while maintaining key characteristics, pooling layers strengthen the model against changes in smoke and fire patterns. By randomly deactivating neurons during training, dropout-enhanced fully connected layers avoid overfitting and guarantee that the model stays generic and independent of particular patterns. The model’s efficiency is greatly increased by the preprocessing stage, which includes color properties and contour analysis. The preprocessing lowers the input size and computational effort by separating Regions of Interest (ROIs) in the pictures, which enables the CNN to concentrate on the most pertinent regions for smoke and fire detection. The drawbacks of traditional techniques, notably high false-positive rates from visually similar things like yellow leaves or brilliant sunlight reflections, are addressed by this hybrid approach. The concatenated CNN improves accuracy and reliability by fusing the advantages of deep learning with preprocessing.

Overall, the suggested system is a major advancement in smoke and fire detection technology. It is a dependable option for practical uses, including early warning systems, safety monitoring, and surveillance, because of its capacity to recognize complex patterns and discriminate between fire, smoke, and things that resemble fire. A new benchmark for fire and smoke detection systems is set when the concatenated CNN is integrated with preprocessing approaches to guarantee that the model achieves high speed, efficiency, and accuracy.

## 4. Experiments and Discussion

### 4.1. Evaluation Metrics

The accuracy, robustness, and computing efficiency of the suggested concatenated CNN model for fire and smoke detection were assessed using several common assessment metrics. These parameters are crucial for evaluating how well the system can differentiate between smoke, fire, and non-fire elements in a variety of environmental settings.

The performance of fire and smoke detection models is evaluated using evaluation measures such as accuracy, precision, recall, and F1-score. Each statistic is crucial for guaranteeing the model’s dependability in practical applications and highlights distinct facets of its performance. An extensive examination of these measures, highlighting their significance in smoke and fire detection systems, is provided below.

The percentage of accurately classified cases—including fire, smoke, and non-fire—out of all cases is known as accuracy. It is computed using the following formula:

$$Accuracy=\frac{TP+TN}{TP+FP+FN+TN}$$

where false positives (FPs) are non-fire elements that were incorrectly classified as fire or smoke, false negatives (FNs) are instances of fire or smoke that the model missed, true positives (TPs) are fire or smoke that was correctly detected, and true negatives (TNs) are non-fire regions that were correctly identified. High accuracy is a useful general performance statistic since it shows that the model can properly categorize most occurrences. However, accuracy by itself may be deceptive in datasets with class imbalances. In a dataset that is primarily composed of non-fire photos, for example, a model that predicts everything as “non-fire” may achieve high accuracy yet be unable to detect fire or smoke when it occurs. For a more thorough understanding of model performance, accuracy must be combined with other metrics, even if it offers a broad perspective.

The accuracy of fire or smoke forecasts is the main emphasis of precision. Using the following formula, it determines the percentage of real positive detections among all cases that are categorized as fire or smoke:

$$Precision=\frac{TP}{TP+FP}$$

A model with high precision reduces false alarms, which is important in real-world applications because needless alerts might result in resource waste, panic, or inefficient operations. Maintaining high precision, for instance, guarantees that only real cases of fire or smoke set off alarms in industrial safety systems or extensive monitoring networks, preventing disruptions brought on by false positives.

Recall, sometimes called sensitivity, gauges how well a model can identify real-world fire or smoke incidents. It is described as follows:

$$Recall=\frac{TP}{TP+FN}$$

A high recall value reduces the possibility of missed detections by guaranteeing that the model recognizes most of the fire or smoke incidents. This is especially important in safety-critical settings where failing to identify a fire or smoke could have disastrous results, including property or human casualties. However, in situations where precision is just as crucial, a model with a high recall may also produce more false positives.

To summarize, recall concentrates on catching all actual fire or smoke incidents, precision guarantees that a model avoids false alarms, and accuracy offers a broad indicator of performance. When combined, these indicators offer a thorough framework for evaluation, guaranteeing the dependability and resilience of smoke and fire detection systems in a variety of demanding settings.

### 4.2. Performance Evaluation and Comparative Analysis

**Figure 5:**
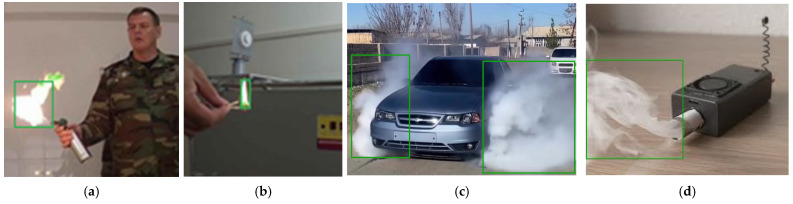
Laboratory experiments: (**a**) large fire; (**b**) small fire; (**c**) multiple smoke; (**d**) single and weak smoke.

The proposed fire and smoke detection method was tested on a diverse sample of eleven videos containing various scenes, including both daytime and nighttime settings, as well as indoor and outdoor environments. These videos were selected to evaluate the robustness and accuracy of the detection system under varying conditions. The experiments aimed to compare the performance of the concatenated CNN model with traditional image processing algorithms. The results are summarized in Table 2, which highlights the performance metrics and key observations from the experiments. The experimental results demonstrated the operational conditions of the proposed model. Frames from laboratory experiments were analyzed to validate the functionality of the system under controlled and real-world conditions, as shown in Figure 5. Instance evidence of the concatenated CNN model’s performance on the D-Fire dataset is shown in Figure 5a—large fire detection, Figure 5b—small fire detection, Figure 5c—combined multiple smoke in a car, and Figure 5d—a weak smoke scenario.

The proposed system consistently detected fire and smoke with higher precision compared to traditional methods. The fourth column of Table 2 specifically addresses errors related to the false-alarm rate (FAR). FAR errors occurred when the system falsely detected fire or smoke in video frames where neither was present. These errors were significantly reduced in the proposed concatenated CNN model compared to the color and contour-based methods. This improvement highlights the model’s ability to minimize false positives, which is crucial for reducing unnecessary alerts and improving system reliability.

The last column of Table 2 presents the overall performance of the concatenated CNN model. The results indicate that the model achieves much higher accuracy in detecting fire and smoke compared to traditional color and contour techniques. This is attributed to the model’s ability to integrate features from both visual (color) and structural (contour) analyses while leveraging the hierarchical feature extraction capabilities of the CNN. The experimental results confirm that the proposed hybrid method, which combines the concatenated CNN model with image processing techniques, is highly effective for fire and smoke detection. The system not only improves accuracy but also significantly reduces the false-alarm rate, making it a reliable solution for diverse real-world applications. This advancement marks a significant step forward in fire and smoke detection technology.

The experimental results demonstrate how well three methods—color characteristics analysis, contour analysis, and the suggested concatenated CNN model—perform in detecting fire and smoke in a variety of settings. The accuracy and false-alarm rate (FAR) in situations with different levels of complexity, including indoor and outdoor environments, day and night lighting, and dynamic conditions, were the main focus of the evaluation. With an accuracy of over 99% in every case, the concatenated CNN model continuously beat both color characteristics and contour analysis. Despite their relatively high accuracy (92.3% to 97.8%), color characteristics and contour analysis performed poorly in situations where fire-like elements—like brilliantly colored objects—introduced visual uncertainty. For instance, the concatenated CNN maintained an accuracy of 99.2% in the “yellow-red clothes” scenario, whereas color characteristics and contour analysis achieved 95.1% and 96.2%, respectively.

Since the concatenated CNN model consistently generated fewer false alarms in all scenarios, the FAR further confirms its superiority. For example, the concatenated CNN exhibited an FAR of only 12 in the “candle with dark background” scenario, whereas both color characteristics and contour analysis showed FARs of 17. In a similar vein, the concatenated CNN reduced false positives while retaining high accuracy in more complicated situations, such as “burning cars” and “on highway”, guaranteeing dependable detection performance under trying circumstances. The concatenated CNN model’s resilience is demonstrated by its capacity to manage a variety of situations. It performs exceptionally well both indoors (like “in kitchen” with 99.5% accuracy) and outdoors (like “in forest” with 99.7% accuracy). Additionally, it is able to adjust to different lighting situations with ease, attaining 99.6% accuracy in “candle with dark background” and 99.4% accuracy in “night lamp and fire”. Its accuracies of 99.2% and 99.4% in processing dynamic scenarios like “explosion” and “burning cars”, respectively, further illustrate its dependability.

The approaches’ advantages and disadvantages show that, although color characteristics analysis work well in situations where the fire colors are different, it is prone to false positives in situations where the colors are similar, such as “yellow-red clothes”. Contrarily, contour analysis is good at identifying structural characteristics but has trouble in situations when contours overlap, such as “in supermarket”. By combining the characteristics of the two methods, the concatenated CNN model gets over these restrictions and guarantees excellent accuracy and a low FAR.

The suggested concatenated CNN model outperforms state-of-the-art techniques for smoke and fire detection, as shown by its improved performance metrics, wider detection range, and versatility in a variety of situations.

**Figure 6:**
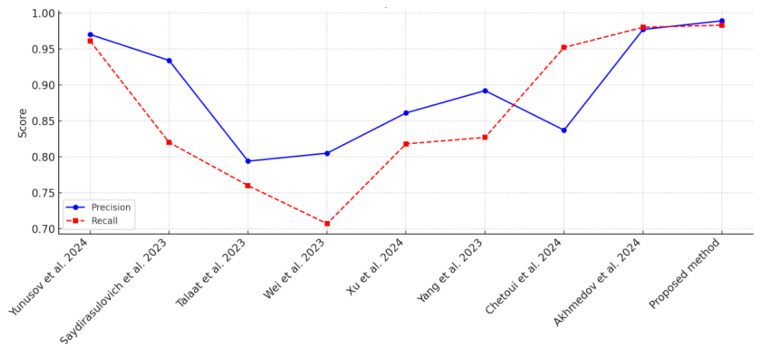
Performance comparison of CCNN, [42,43,44,45,46,47,48,49].

In Table 3 and Figure 6, several fire and fire/smoke detection models are compared according to their precision, recall, dataset size, and object type. Outperforming all other models, the suggested approach, which makes use of a concatenated CNN, has the greatest accuracy (0.989) and recall (0.983). The bigger dataset utilized in the study (21,527 photos) allowed for higher generalization and resilience, which is why it performed better. The fact that it can detect smoke and fire further demonstrates how versatile it is in a variety of real-world situations.

The second-best performance among YOLO-based models was provided by Akhmedov et al. [49] using YOLOv10m, who used 9235 pictures devoted to fire detection to achieve an accuracy of 0.977 and a recall of 0.98. In a similar vein, Yunusov et al. [42] showed that YOLOv8 is effective in managing intermediate dataset sizes (7000 photos), with an accuracy of 0.97 and a recall of 0.911. The somewhat poorer recall in comparison to YOLOv10m, however, indicates that model setup and dataset size are critical factors in striking a balance between accuracy and recall. In order to obtain competitive results, Chetoui et al. [48] and Yang et al. [47] used YOLOv8 and YOLOv5, respectively, in both fire and smoke detection tasks. With 11,667 photos, Chetoui et al. achieved a recall of 0.952, demonstrating the model’s multi-object identification capabilities. With the same dataset size, Yang et al. achieved an accuracy of 0.892 and a recall of 0.827, showing balanced performance on both criteria. These models show how YOLO architectures may be tailored to intricate object identification tasks that include several classes.

Wei et al. [45] with YOLOv8 and Saydirasulovich et al. [43] with YOLOv6 are examples of lower-performing models demonstrating how model settings and dataset size affect detection performance. While Wei et al. demonstrated the lowest recall (0.707) with just 2059 photos for fire detection, Saydirasulovich et al. used 4000 images and obtained a precision of 0.934 but a recall of only 0.82. These findings highlight how a model’s capacity to generalize can be restricted by fewer datasets, which can result in greater false-negative rates. Because of its large dataset and sturdy design, the suggested concatenated CNN model is the most successful in terms of both precision and recall. Strong performance is also shown by YOLO-based models, specifically YOLOv10m and YOLOv8, especially when combined with enough training data. In order to achieve high-performance fire and fire/smoke detection, the analysis emphasizes the significance of dataset size, model design, and flexibility in multi-object detection.

The performance of fire and smoke detection models is evaluated using metrics such as accuracy, precision, recall, and F1-score. Table 3 compares the proposed concatenated CNN with YOLO-based models across diverse datasets, providing a benchmark of its generalizability. However, recognizing potential variability due to dataset composition, we note that precision and recall values are context-specific, influenced by factors such as annotation quality and object diversity. To address this, Table 4 presents an intra-dataset analysis, comparing performance on a 10,000-image subset and the full 21,527-image D-Fire dataset, confirming the model’s robustness. This dual approach enhances the interpretability of the inter-dataset comparison in Table 4.

The performance of several smoke and fire detection models evaluated on the D-Fire dataset demonstrates the remarkable potential of the suggested concatenated CNN model in contrast to cutting-edge methods, as shown in Table 5. The evaluation criteria, accuracy and recall, offer a thorough analysis of each model’s capacity to consistently and precisely identify smoke and fire in a variety of settings.

**Figure 7:**
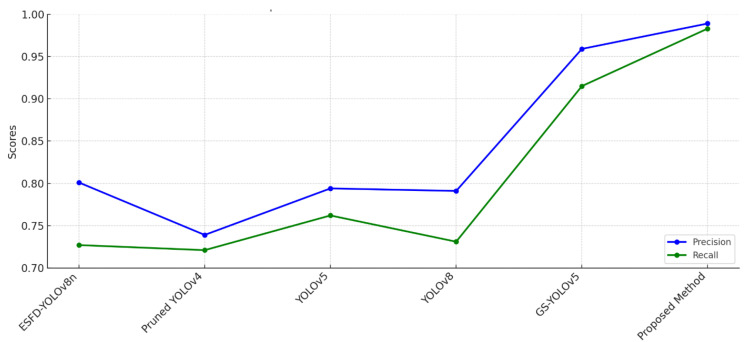
Performance comparison of CCNN model.

The suggested concatenated CNN model outperformed all the other models by achieving the highest accuracy (0.989). This high precision shows that the algorithm can efficiently minimize false positives while properly classifying fire and smoke incidents. Figure 7 illustrates that the accuracy of 0.959 obtained by Chen et al. [54] using GS-YOLOv5 is the closest to the suggested approach. However, GS-YOLOv5’s evaluation is limited by the lack of recall data, raising questions about the model’s overall dependability. A larger proportion of false positives is suggested by the much lower accuracy of other models, such as Mamadaliev et al. [50] using ESFD-YOLOv8n (0.801 precision) and Peng et al. [53] with YOLOv8 (0.791 precision). The lowest accuracy values (0.739 and 0.794) are displayed by models such as Venancio et al.’s [51,52], who used Pruned YOLOv4 and YOLOv5, further highlighting their shortcomings in correctly differentiating between smoke and fire.

The concatenated CNN model outperformed the other models with an astounding recall of 0.983. In safety-critical situations where missed detections might have dire repercussions, high recall guarantees that the model identifies almost all fire and smoke incidents while reducing false negatives. By contrast, Mamadaliev et al. [50] with ESFD-YOLOv8n obtained a recall of 0.727, whereas Peng et al. [53] with YOLOv8 achieved a recall of 0.731. These models’ comparatively poor memory values suggest that they would regularly overlook real-world fire or smoke incidents, which would diminish their dependability in practical settings. It is challenging to thoroughly assess the models of Venancio et al. [51,52] and Chen et al. [54] for their capacity to reduce false negatives because their recall values were sadly not disclosed.

On the D-Fire dataset, which is renowned for its complexity and variety, the suggested concatenated CNN model further exhibits exceptional generalization capacity. The concatenated CNN uses a hybrid architecture that incorporates preprocessing methods like color characteristics and contour analysis, whereas models like YOLOv8 and ESFD-YOLOv8n only use end-to-end deep learning. By refining the input characteristics, these preprocessing processes enable the model to perform better and discriminate fire and smoke from visually identical elements. The YOLO-based models, on the other hand, are more likely to produce false positives and false negatives because they lack preprocessing refinement, even though they are successful in real-time object recognition.

The trade-offs between recall and accuracy in various models are clearly highlighted by the comparison. The absence of recall data raises questions regarding whether Chen et al. [53] compromised detection completeness (more false negatives) in order to achieve lower false positives, despite the model’s comparatively high accuracy of 0.959. In a similar vein, models such as Peng et al.’s [51] and Mamadaliev et al.’s [50] have poor recall values, which reduces their dependability for comprehensive fire and smoke detection. These drawbacks highlight the concatenated CNN model’s resilience, which achieves a balance between high precision and recall.

The suggested concatenated CNN model demonstrates itself as the best-performing solution for fire and smoke detection on the D-Fire dataset. Its remarkable dependability in reducing false positives and false negatives is demonstrated by its capacity to attain a 0.989 accuracy and a 0.983 recall. It overcomes the drawbacks of YOLO-based models by combining preprocessing methods with deep learning, creating a more reliable solution for actual fire and smoke detection applications. The concatenated CNN model is a very useful and efficient tool for safety and surveillance applications because of these results, which establish a new standard in the area.

## 5. Conclusions

Using the D-Fire dataset, this paper provides a unique concatenated CNN model for fire and smoke detection that is thoroughly tested against both conventional and cutting-edge techniques. The suggested approach combines a deep learning framework with hybrid preprocessing methods, such as color characteristics analysis and contour-based algorithms, to efficiently extract and combine data for better performance in smoke and fire detection tasks. The experimental findings show that the concatenated CNN model outperforms both sophisticated YOLO-based models and conventional techniques, achieving unmatched accuracy (0.989) and recall (0.983). The suggested hybrid approach uses preprocessing to refine Regions of Interest (ROIs), lowering false negatives in complex environments and false positives from fire-like objects, in contrast to existing models that either only concentrate on fire detection or mainly rely on end-to-end deep learning pipelines. Along with pooling layers that increase computing efficiency and durability, the design also includes fully linked layers with dropout to guarantee generalization.

Novelties of the proposed methodology include the following:

- Hybrid Design: Combines preprocessing techniques (color characteristics and contour analysis) with a deep learning framework, addressing limitations of both traditional and end-to-end deep learning methods.
- Dual Capability: Detects both fire and smoke, making it more versatile than existing methods that focus solely on fire detection.
- Dataset Generalization: Demonstrates robustness across diverse scenarios in the D-Fire dataset, including varying lighting conditions, dynamic environments, and overlapping fire and smoke features.
- Error Reduction: Achieves a significantly lower false-alarm rate (FAR) and a high accuracy compared to other models, minimizing the likelihood of false positives and missed detections.

The suggested technique has drawbacks even if it sets a new standard for smoke and fire detection technologies. Compared to strictly end-to-end models like YOLOv5 or YOLOv8, the hybrid preprocessing phase adds more computational cost even though it is essential for improving accuracy. Additionally, even though the model performs exceptionally well in terms of accuracy and recall, its dependence on a supervised learning framework requires annotated datasets, which might not be easily accessible for all types of smoke and fire investigations. A future study might examine decreasing computing complexity and broadening the approach to handle real-time applications more successfully.

The performance of the proposed concatenated CNN model depends heavily on the quality and accuracy of the training data. However, obtaining accurately described training data can be challenging, particularly for specific scenarios such as rare fire events or complex environments. For example, fire and smoke events are relatively rare, making it difficult to collect a large and diverse dataset. Additionally, manually annotating fire and smoke regions in images and videos is time-consuming and prone to errors, especially in complex scenarios where fire and smoke may overlap with other objects or vary in appearance due to lighting conditions.

To address these challenges, several potential solutions can be explored. Data augmentation techniques, such as rotation, flipping, and scaling, can be used to artificially increase the size and diversity of the training dataset. Synthetic data generation, using computer graphics or simulation tools, can supplement real-world data by creating realistic fire and smoke images in various environments. Semi-supervised learning approaches can leverage unlabeled data to improve model performance, reducing the reliance on accurately annotated datasets. Finally, collaborative data collection efforts with organizations such as fire departments or industrial facilities can help gather real-world fire and smoke data in diverse scenarios.

Future work will focus on implementing these solutions to improve the quality and diversity of training data, ensuring that the proposed model performs well in specific and challenging scenarios.

In summary, the suggested concatenated CNN model offers a reliable, accurate, and adaptable solution for safety-critical applications, greatly enhancing fire and smoke detection capabilities. Notwithstanding its drawbacks, the combination of deep learning and hybrid preprocessing makes it a dependable option for settings needing high detection recall and precision, opening the door for more advancement in this crucial field of study.

## Figures and Tables

**Table 1:** Detailed parameters of proposed concatenated CNN.

Layer	Input 1	Input 2
**First convolution layer**	3 × 3 kernel, 64 filters, and ReLU activation	3 × 3 kernel, 64 filters, and ReLU activation
**Pooling layer**	Stride = 2, 2 × 2 pooling	Stride = 2, 2 × 2 pooling
**Second convolution layer**	3 × 3 kernel, 128 filters, and ReLU activation	3 × 3 kernel, 128 filters, and ReLU activation
Pooling layer	2 × 2 pooling	2 × 2 pooling
**Third convolution layer**	3 × 3 kernel, 256 filters, and ReLU activation	3 × 3 kernel, 256 filters, and ReLU activation
**Pooling layer**	2 × 2 pooling, which yields a 64 × 64 × 256 feature map	2 × 2 pooling, which yields a 64 × 64 × 256 feature map
**Concatenated layer**	Along the final axis, concatenate the feature maps from the two branches	
	64 × 64 × 768 is the final feature map size, 256 from each branch	
Fourth convolution layer	3 × 3 kernel, 512 filters, and ReLU activation	
**Pooling layer**	2 × 2 pooling, which makes the feature map 32 × 32 × 512 in size	
**Fifth convolution layer**	3 × 3 kernel, 1024 filters, and ReLU activation	
**Pooling layer**	2 × 2 pooling, making the feature map 16 × 16 × 1024 in size	
**Flatten layer**	Create a 1D vector by flattening the 3D feature map	
**First dense layer**	1024 neurons, activation of the ReLU	
First dropout layer	Dropout rate = 0.5	
Second dense layer	ReLU activation and 512 neurons	
Second dropout layer	Dropout rate = 0.5	
**Output**	Probabilities are assigned by Softmax activation for two neurons (fire, smoke, or non-fire)	

**Table 2:** Fire and smoke recognition results.

Description of Fire and Smoke	Number of Frames	Fire and Smoke Frames	Color Characteristics		Contour Analysis		Concatenated CNN
			FAR	Accuracy	FAR	Accuracy	
Candle with dark background	1906	876	17	96.8	12	97.2	99.6
Day lamp and fire	2513	1562	22	93.5	16	94.8	98.9
Night lamp and fire	591	263	8	97.8	4	96.3	99.4
In kitchen	1255	790	14	95.7	11	96.1	99.5
In supermarket	785	560	9	96.2	10	95.7	99.6
In forest	1710	1026	17	94.7	13	94.9	99.7
In mountain	2045	1210	28	94.1	22	94.9	99.5
Yellow-red clothes	1652	980	34	95.1	23	96.2	99.2
On highway	1680	816	21	92.3	19	92.4	99.1
Explosion	425	214	4	95.2	7	94.6	99.2
Burning cars	870	570	11	94.9	15	94.3	99.4

**Table 3:** Comparison with YOLO-based models.

References	Model	Precision	Recall	Images	Object
Yunusov et al. [42]	YOLOv8	0.97	0.961	7000	Fire
Saydirasulovich et al. [43]	YOLOv6	0.934	0.82	4000	Fire/smoke
Talaat et al. [44]	YOLOv8	0.794	0.7.6	6000	Fire/smoke
Wei et al. [45]	YOLOv8	0.805	0.707	2059	Fire
Xu et al. [46]	YOLOv7	0.861	0.818	2058	Fire
Yang et al. [47]	YOLOv5	0.892	0.827	11,667	Fire/smoke
Chetoui et al. [48]	YOLOv8	0.837	0.952	11,667	Fire/smoke
Akhmedov et al. [49]	YOLOv10m	0.977	0.98	9235	Fire
Proposed method	Concatenated CNN	0.989	0.983	21,527	Fire/smoke

**Table 4:** Intra-dataset performance of the concatenated CNN on subsets of the D-Fire dataset.

Dataset Size	Training Images	Validation Images	Test Images	Precision	Recall	Notes
10,000	7000	1500	1500	0.987	0.981	Subset of D-Fire
21,527	15,069	3229	3229	0.989	0.983	Full D-Fire

**Table 5:** Performance comparison with different models in the same dataset.

References	Model	Precision	Recall	Dataset
Mamadaliev et al. [50]	ESFD-YOLOv8n	0.801	0.727	D-Fire
Venancio et al. [51]	Pruned YOLOv4	0.739	0.721	
Venancio et al. [52]	YOLOv5	0.794	0.762	
Peng et al. [53]	YOLOv8	0.791	0.731	
Chen et al. [54]	GS-YOLOv5	0.959	0.915	
Proposed method	Concatenated CNN	0.989	0.983	

## Data Availability

Data are contained within the article.
